# Tailored performance dashboards—an evaluation of the state of the art

**DOI:** 10.7717/peerj-cs.625

**Published:** 2021-10-05

**Authors:** Artem Kruglov, Dragos Strugar, Giancarlo Succi

**Affiliations:** Innopolis University, Innopolis, Russia

**Keywords:** Software engineering, Adaptable dashboards, Software process management, Literature review

## Abstract

**Context:**

Tailoring mechanisms allow performance dashboards to vary their appearance as a response to changing requirements (e.g., adapting to multiple users or multiple domains).

**Objective:**

We analyze existing research on tailored dashboards and investigate different proposed approaches.

**Methodology:**

We performed a systematic literature review. Our search processes yielded a total of 1,764 papers, out of which we screened 1,243 and ultimately used six for data collection.

**Results:**

Tailored dashboards, while being introduced almost thirty years ago, did not receive much research attention. However, the area is expanding in recent years and we observed common patterns in novel tailoring mechanisms. Since none of the existing solutions have been running for extended periods of time in real-world scenarios, this lack of empirical data is a likely cause of vaguely described research designs and important practical issues being overlooked.

**Implications:**

Based on our findings we propose types of tailoring mechanisms taking into account the timing and nature of recommendations. This classification is grounded in empirical data and serves as a step ahead to a more unifying way of looking at tailoring capabilities in the context of dashboards. Finally, we outline a set of recommendations for future research, as well as a series of steps to follow to make studies more attractive to practitioners.

## Introduction

Information dashboards offer a unique way of gaining insights from existing information, and are key for extracting knowledge in different contexts. According to Few (2006), Few (2004) and Few & Edge (2007), they are defined as a visual display of the most important information needed to achieve certain objectives; consolidated and arranged on a single screen so the information can be monitored at a glance. Information dashboards are widely used in many different domains as noted by Sarikaya et al. (2018), one of which is optimizing business processes. Such dashboards are often referred to as *performance dashboards*.

### Performance dashboards

Performance dashboards enable organizations to measure, monitor, and manage business performance more effectively Eckerson (2010). They build on foundations of business intelligence and data integration infrastructure, and are used for monitoring, analysis, and management. The most popular framework for developing such dashboards was introduced in Eckerson (2010) where the author proposes a set of questions that serve as guidelines for dashboard architecture engineering. Ideas presented in this book have been discussed and used in practise extensively.

However, some of the guidelines outlined in the book assume a particular set of technologies that is not popular nowadays, such as multimedia plugins. Web development has evolved drastically since then, which makes these questions ineffective at guiding the dashboard architect in the right direction.

### Tailored dashboards

In addition to the technical difficulties described above, dashboard architects face an even harder problem: how to structure and display relevant information in a way that stands the test of time. Software companies used agile and lean methods to do just this: to welcome changing requirements and to respond to these changes Beck et al. (2001) and Janes & Succi (2014). Changing requirements can take many shapes and forms, but in the context of dashboards they usually amount to tailoring the dashboard to every user. Dashboards that can vary their appearance and functionalities to match the users’, data’s and context’s requirements are referred to as tailored dashboards in Vázquez-Ingelmo, García-Peñalvo & Therón (2019b) and we use this term throughout the paper.

Since performance dashboards are used by people holding different positions in the company, and having different goals, this task becomes increasingly harder in the sense that each user requires a different set of metrics. As it is infeasible to tailor the dashboard to each and every user in the organization individually, personalized, customizable, and adaptive dashboards are continuing to gain traction, as described by Vázquez-Ingelmo, García-Peñalvo & Therón (2019). Widely popular options include Tableau (https://tableau.com/) and Grafana (https://grafana.com/), both of which require no programming skills for users to customize their dashboards themselves. While easy to use and easily customizable, these solutions are as effective as users make them to be. Put differently, users might not know which configuration best matches their goals, as explained by Padilla et al., (2018). This is the motivation behind some of the more sophisticated methods of dashboard design.

### Existing reviews

The research in the area of tailored dashboard design has been summarized by Vázquez-Ingelmo, Garcia-Peñalvo & Therón (2019a). They categorized already proposed techniques in terms of the tailoring method in five categories: customizable, customizable with system support, personalized, hybrid, and adaptive. Customizable dashboards involve approaches that require manual intervention and explicit user requirements for selecting visual displays. Personalized solutions are the ones that infer a configuration from existing data about users and their goals, but can not be modified at run-time. Adaptive (adaptable) solutions, on the other hand, restructure themselves based on user-system interactions. Hybrid approaches are the ones that do not fit in either one of these categories, or combine ideas from multiple ones.

Even though the research and development in the field of performance dashboards is constantly growing, as noted by Vázquez-Ingelmo, García-Peñalvo & Therón (2019b), the last comprehensive literature review on the topic, to the best of our knowledge, was written in 2012 by Yigitbasioglu & Velcu (2012). They covered most of the fundamental theory and rationale behind performance dashboards, dating back to early 1990s, including decision support systems. They also explored to what extent should users’ knowledge, education, experience, skills, and cognitive types influence the dashboard design process. In addition, they compiled down a diagram that outlines a research path with implications on dashboard design. However, they did not focus on existing approaches, partly because most of them have been proposed after this review has been published.

### Goals and contributions

Past research in this area either focused on tailored dashboards, or domain-specific functionalities for different types of dashboards, not necessarily performance ones. The outcome of the systematic review helps identify further research directions when it comes to developing tailored performance dashboards specifically, though. As noted previously and in Vázquez-Ingelmo, Garcia-Peñalvo & Therón (2019a), there are different approaches when it comes to tailoring capabilities, and most of these have been applied in particular domains that is not performance monitoring. Therefore, it is crucial to identify if any of the approaches that have been used already are easily transferable to performance dashboards. In addition, we seek to provide rationale that would explain which of the tailoring approaches would best fit the domain of performance monitoring.

We find that the results of this review could be particularly useful for practitioners aiming to design new performance dashboard solutions with tailoring capabilities. In addition, researchers could use the proposed possible future research opportunities to close some of the gaps in the field of tailored dashboards. For reference, we outline how both parties could benefit from our findings in a form of table, separated by our research questions. We also include guidelines for future researchers who wish to make their studies more attractive to practitioners.

### Paper structure

In ‘Research Method’ we discuss the review process we used to avoid bias and ensure replicability. ‘Results’ outlines the demographic information we collected and the results obtained following the analysis and data extraction. An interpretation of the results takes place in ‘Discussion’. In ‘Threats to Validity’ we present some of the limitations of our review, and ‘Conclusions’ concludes the paper.

## Research Method

**Figure 1:**
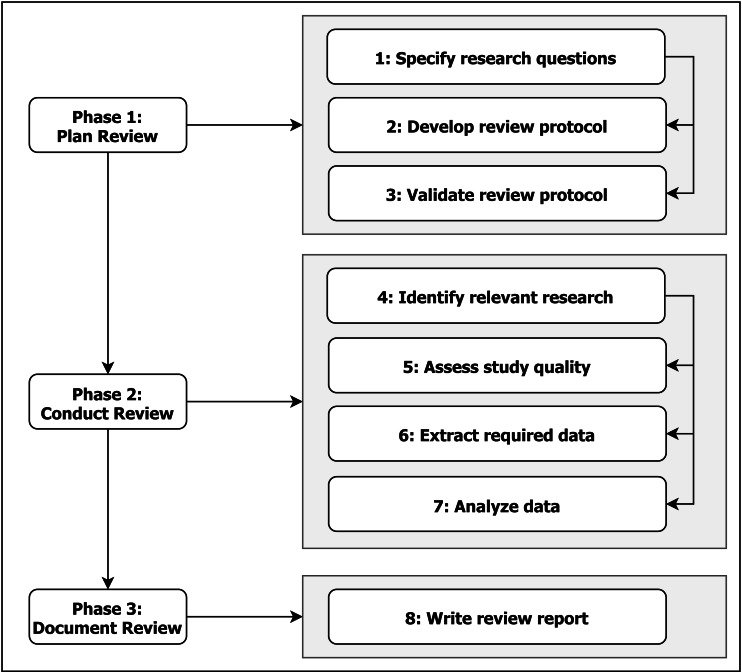
SLR Process (adapted from Brereton et al. (2007)).

The systematic literature review (SLR) follows the overall the process proposed by Kitchenham (2004) and Kitchenham & Charters (2007); moreover, in terms of detailed process we have adhered to the recommendations of Brereton et al. (2007), as detailed in Fig. 1. In this section we describe how we carried it out and the steps taken to ensure a fair, credible, and unbiased evaluation of existing approaches in the area of tailored performance dashboards following the mentioned works. The structure of our review has also been influenced by Vázquez-Ingelmo, Garcia-Peñalvo & Therón (2019a), as they conducted a more general review in the field, but still very closely related.

### Review planning

In this stage of the review process from the very start we wanted to ensure rigor and make our results repeatable. After defining a set of research questions, we outline our review protocol, and include details to ensure that our results are reproducible. This includes exclusion and inclusion criteria as well as the search process. Moreover, we added a quality assessment layer on top. Based on our research questions, we defined keywords and search strings, after which we proceeded to collect the papers and review them.

#### Research Questions

To start off, we used a widely popular Goal Question Metric (GQM) paradigm to obtain our questions, as suggested by Basili, Caldiera & Rombach (1994):

• **Purpose**: *analyze and characterize;*
• **Issue**: *handling of tailoring capabilities;*
• **Object**: *in performance dashboards;*
• **Viewpoint**: *from a researcher’s point of view.*

Based on our goal, we obtained the following research questions:

• **RQ1.** Have tailoring capabilities ever been applied to performance dashboards in the past?
• **RQ2.** Which factors should be taken into account when designing tailoring capabilities specifically for performance dashboards (requirement management)?
• **RQ3.** Given that tailored dashboards are often prone to the cold-start problem, what are some of the approaches and/or ideas to help combat it?
• **RQ4.** How effective would tailored performance dashboards be after the users have used the system for a long period of time?

With the first three research questions we seek to identify the methods that have been, or could be used to design user-tailored functionalities in performance dashboards. The fourth question deals with the sustainability of such software systems.

RQ1 aims to find any existing solutions. It would fit better in the systematic mapping rather than a review, but it is included here because we will both analyze and interpret what these results would mean. RQ2 delves deeper into the topic by asking what tailoring method would be most effective for performance dashboards and how they work in terms of their requirement engineering method. Cold-start problem is known to cause inaccurate recommendations in tailoring systems Schein et al. (2002) and Lika, Kolomvatsos & Hadjiefthymiades (2014) and therefore finding an effective way to combat it is crucial. RQ3 tries to identify how this problem could be solved. Finally, RQ4 deals with sustainability of such software systems.

We also defined a set of PICO criteria initially advocated by Petticrew & Roberts (2008) to narrow down the research scope:

• **Population:** existing software solutions and theoretical frameworks;
• **Intervention:** tailoring mechanisms that can be applied to performance dashboards;
• **Comparison:** N/A;
• **Outcomes:** methods and mechanisms for achieving tailored functionalities.

Please note that the scope is narrowed down to either tailored performance dashboards specifically, general tailored dashboards that can be applied to performance monitoring, or tailored dashboards in different domains that have a clearly defined procedure for adapting to different domains. Our decision to include non-performance tailoring dashboard stems from the observation that if the transition requires low effort, it could be worth porting this approach to performance monitoring.

#### Review protocol

With research questions formulated, we proceed to explain the details of the review protocol development and validation. One guiding factor was to remain unbiased throughout the process. We meant to achieve this by being completely transparent with the raw data and factors that influenced the decision on whether or not a paper will ultimately be included.

Identifying a set of Exclusion Criteria (EC) as well as Inclusion Criteria (IC) to further narrow down papers relevant to our research questions was the next step. A paper has to fulfill all the IC and not meet any of the EC to be included in our review.

• **EC1:** the work is not written in the English;
• **EC2:** the paper does not describe a software solution or a theoretical software solution framework;
• **EC3:** the solution’s context are neither tailored performance dashboards, general tailored dashboards that can be applied to performance monitoring, nor tailored dashboards in non-performance domains where authors explicitly mention that the solution can be ported to other domains;
• **EC4:** the paper is not published in peer-reviewed journals, books, or conferences;
• **EC5:** the work does not propose nor discuss possible ways of automatically suggesting metrics to the user.

The inclusion criteria are the opposite of the exclusion criteria:

• **IC1:** the work is written in the English;
• **IC2:** the paper describes a software solution or a theoretical software solution framework;
• **IC3:** the solution’s context are either tailored performance dashboards, general tailored dashboards that can be applied to performance monitoring, or tailored dashboards in non-performance domains where authors explicitly mention that the solution can be ported to other domains;
• **IC4:** the paper is published in peer-reviewed journals, books, or conferences;
• **IC5:** the work either proposes or discusses possible ways of automatically suggesting metrics to the user.

This set of IC and EC guarantees that the review will include solutions applicable to the domain of performance monitoring, which is our goal. What this set does not guarantee is that papers will be solely meant for performance dashboards, primarily because of EC3 and IC3. These two criteria allow non-performance articles and papers to be selected for the review as long as it can be objectively determined if they can be applied to performance dashboards (either they are general so by definition they can be applied to any context, or they are specific to some other domain with a mention from authors that this solution is able to be ported to other domains, in our case performance management). Also note that all selected solutions must have tailoring capabilities.

#### Search strategy

Even though some SLRs in the field are relying on manual search a predefined set of journals, we wanted to get a broader idea of the field of tailored performance dashboards. By going through publications only in several journals and conference proceedings, it is our understanding that we could possibly miss important insights about our research questions (RQs). Therefore, as for our databases for papers we chose some of the most renowned and heavily used ones: Springer, Web of Science (WoS), IEEE Xplore, and Scopus.

Our search queries were heavily influenced by our RQs. These terms are:

• **dashboards**: excluding ones used for health monitoring and in the automotive industry;
• **performance dashboards**: a particular domain of a wide area of dashboards;
• **tailored functionalities**: categories suggested by Vázquez-Ingelmo, Garcia-Peñalvo & Therón (2019a).

#### Query strings

To obtain the query strings, as stated above, we used PICO criteria, connected with Boolean operators *OR*, *NOT* and *AND*. Below we present the search queries we used to fill our initial database of papers, and we present them here to ensure that our results can be repeated and reproduced:

• Scopus: TITLE-ABS-KEY((dashboard*) W/10 (personal* OR perfor* OR adapt* OR flexib* OR tailor* OR context-aware OR generat* OR compos* OR select* OR template*) OR ((dashboard*) AND ((heterogeneous OR different OR diverse OR dynamic) W/0 (requirement* OR stakeholder* OR user* OR need* OR task* OR necess*)))) AND NOT TITLE-ABS-KEY (car OR vehicle OR custom* OR automo* OR driving OR drive OR medic* OR health*) AND NOT DOCTYPE(cr)
• WoS: TS =((meta-dashboard*) OR ((dashboard*) NEAR/10 (personal* OR perfor* OR adapt* OR flexib* OR tailor* OR context-aware OR generat* OR compos* OR select* or template*)) OR ((dashboard*) AND ((heterogeneous OR different OR diverse OR dynamic) NEAR/0 (requirement* OR stakeholder* OR user* OR need* OR task* OR necess*)))) NOT TS = (car OR vehicle OR automo* OR driving OR drive OR medic* OR health*)
• Springer: ^1^ 1We also added an additional restriction for requiring papers to have dashboard* in their titles as SpringerLink scans full-texts of papers for the query strings. Also, we selected English as the language of the study not to manually filter non-English works out.((meta-dashboard*) OR ((dashboard*) NEAR/10 (personal* OR perfor* OR adapt* OR flexib* OR tailor* OR context-aware OR generat* OR compos* OR select* or template*)) OR ((dashboard*) AND ((heterogeneous OR different OR diverse OR dynamic) NEAR/0 (requirement* OR stakeholder* OR user* OR need* OR task* OR necess*))))
• IEEE Xplore: (((meta-dashboard) OR ((dashboard) NEAR/10 (personal* OR perfor* OR adapt* OR flexib* OR tailor OR tailored OR configurable OR context-aware OR generated OR generation OR composed OR composition OR selection OR selecting OR template)) OR ((dashboard) AND ((heterogeneous OR different OR diverse OR dynamic) NEAR/0 (requirement OR stakeholder OR user OR need OR task OR necessities)))) AND NOT (car OR vehicle OR health* OR driving OR drive OR medic*))

Note the use of the Boolean *OR* operator when joining performance monitoring solution and tailoring functionalities. Our rationale for doing this is that we not only want tailored performance dashboarding solutions, but solutions in general domain or non-performance domains that can be ported to the domain of performance dashboards. This has already been discussed previously when we mentioned the inclusion and exclusion criteria. By not using *OR* and instead choosing *AND* we would get only a subset of solutions that could be potentially useful for tailored performance dashboard design.

#### Quality criteria

In addition to the inclusion criteria (IC) and exclusion criteria (EC) we defined previously, we wanted to add an additional step in the selection funnel. Namely, we added quality criteria (QC). The work *quality* here refers to paper’s ability to answer their own research questions. We asked ourselves a standard set of QC questions for each of the papers that went through the process of inclusion and exclusion. These questions are:

• **QC1:** Research questions reflect researchers’ aim to improve functionalities of performance dashboards from the users’ standpoint;
• **QC2:** The authors did not mention major issues or limitations to their research process and results that could harm the effectiveness of tailoring mechanisms.

If a paper satisfied all the IC and did not meet any of the EC criteria, then its quality was evaluated. For QC1, *Yes* or *No* answers were given as a review response for this criterion. For QC2, score was determined on a scale from one to three: one would mean that the authors mentioned major issues or limitations that significantly harm the effectiveness of recommendations. A score of two is assigned to papers where the authors mentioned very minor limitations to their approach that do not influence the recommendations. And finally a score of three would be assigned to works where the authors mentioned no issues whatsoever.

A *No* answer to the QC1 or a score of one to QC2 immediately triggers a removal from the process, causing the paper not to be included in the further review process. A score of two for QC2 would not trigger the removal since it has no influence on the tailoring process.

### Review process

**Figure 2:**
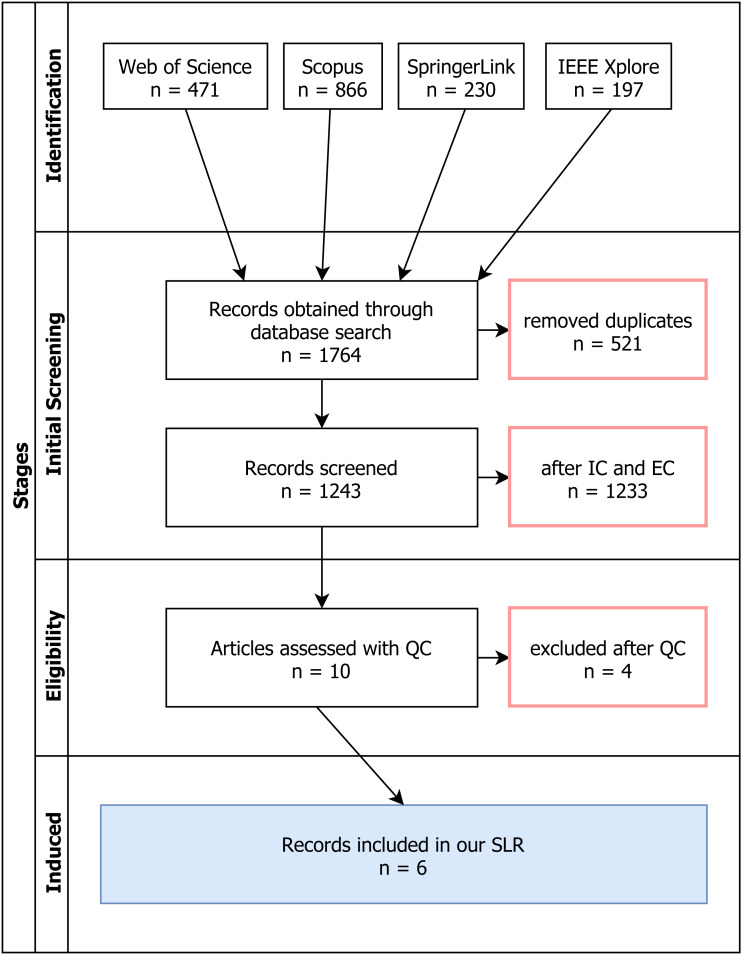
SLR Phases and Outcomes of the Review Process (PRISMA-like).

We used a funnel-like strategy with four major phases in it construct an overall picture of the papers we ended up selecting at the end. To illustrate this, we used a PRISMA-like diagram as suggested by Moher et al. (2009) as can be seen on Fig. 2.

Once we exported all the results from the four aforementioned databases, we created a joint spreadsheet that contained all the records. After performing an initial built-in filter for duplicates, we proceeded to screen the resulting records. This spreadsheet also contains the final results, and is available on this link: http://bit.ly/tailored-perf-slr. In addition, we created a GitHub repository with all the data: https://github.com/d11r/tailored-dashboard-slr.

First we looked for any exclusion criteria (EC), and if any paper satisfied any one of them, we added a note next to the paper that indicates which EC excluded the paper. Note that even if multiple EC hold, one is enough to disqualify the paper and only one EC is noted in the spreadsheet. After the EC and IC have been applied to all the papers, non-marked papers were subject to review with regards to quality criteria (QC). Our QC from a previous sub-chapter were applied and again we marked papers that did not satisfy these conditions. The process yielded a total of six (6) papers.

## Results

**Figure 3:**
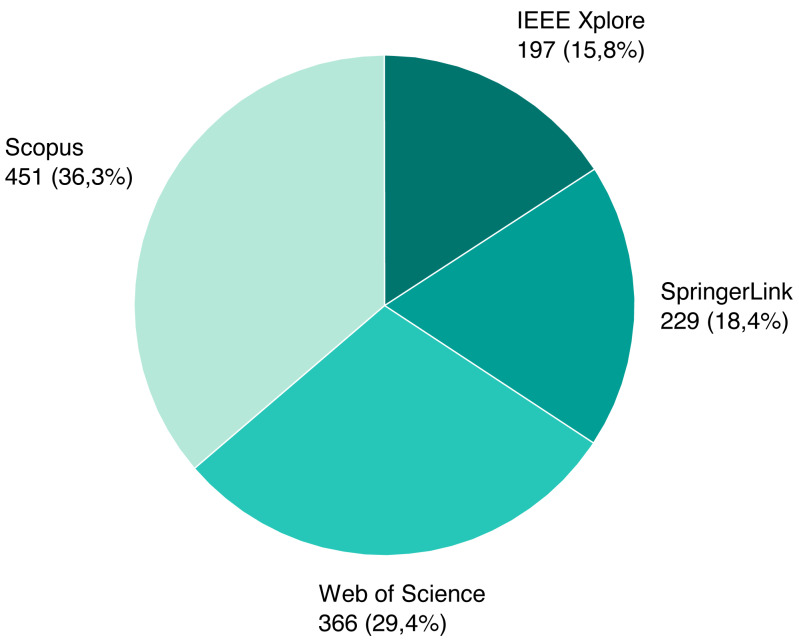
Identified papers per source.

**Figure 4:**
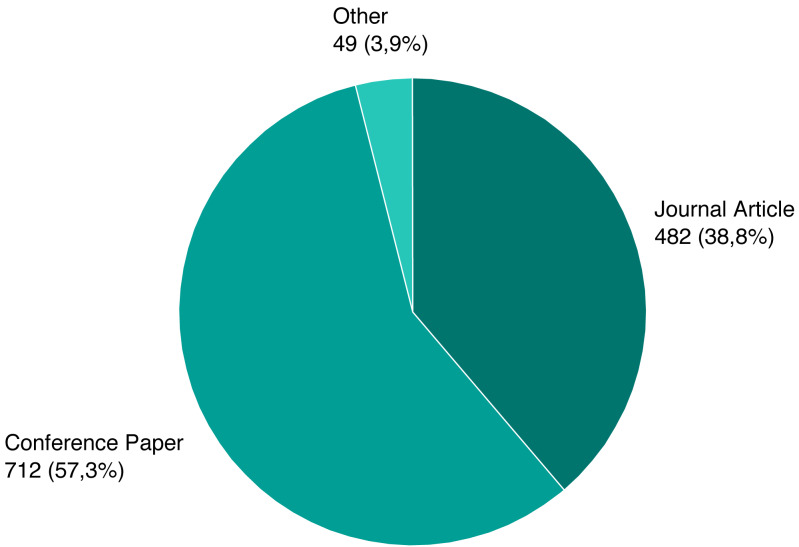
Number of papers per type.

**Figure 5:**
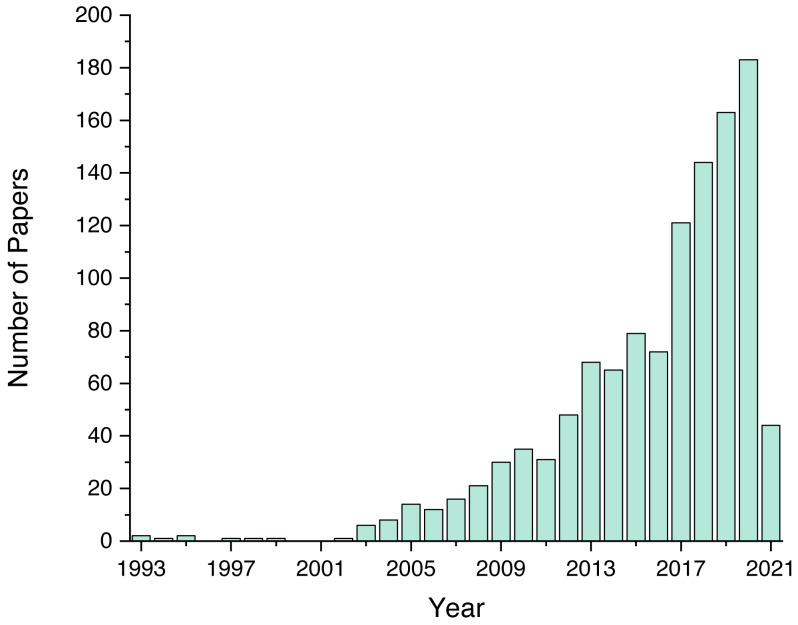
Number of papers per publication year.

As mentioned previously, we screened a total of 1,243 unique papers, and their per-source distribution is depicted on Fig. 3. Figure 4 shows the number and ratio of papers per paper type, and Fig. 5 presents a yearly distribution of papers’ publication years.

**Figure 6:**
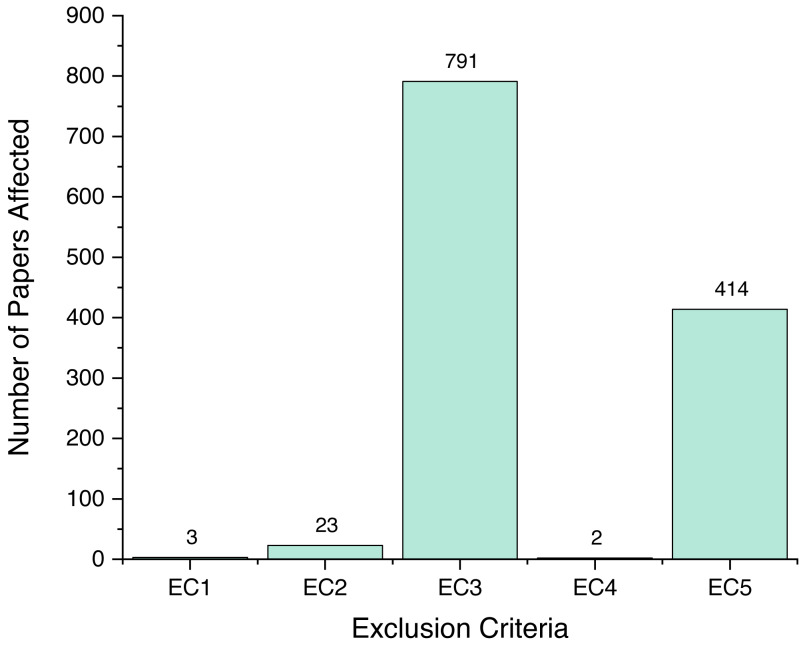
Number of papers affected by EC.

**Figure 7:**
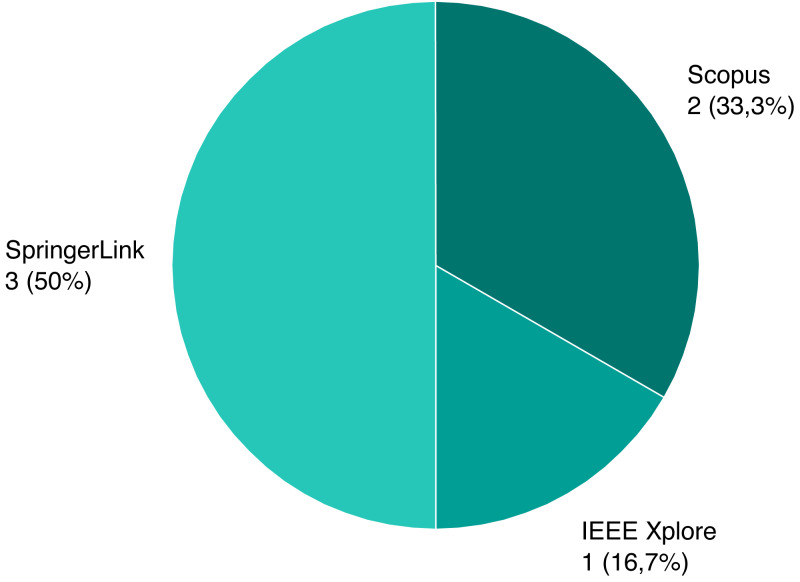
Type distribution of selected papers.

**Figure 8:**
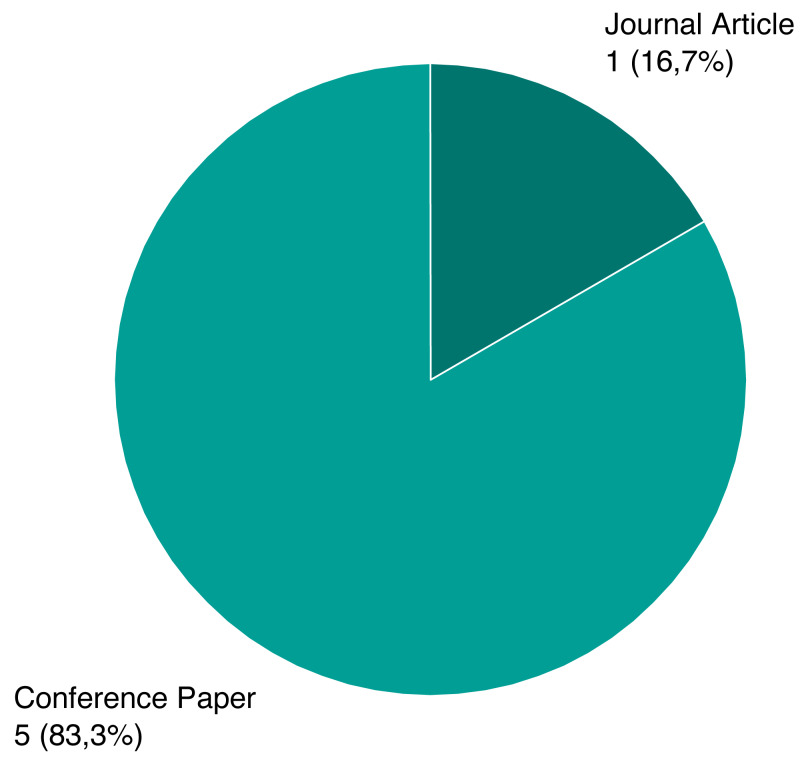
Source distribution of selected papers.

Following the review process, we ended up with six papers, presented on Table 1. We assigned each rejected paper an exclusion criterion that was used for the rejection, and the distribution of these criteria for all rejected papers is presented on Fig. 6. Papers that passed this stage and the quality criteria (QC) are included in our review, and their types and sources are shown on Figs. 7 and 8.

**Table 1:** Studies included in the final review.

**No.**	**Authors**	**Year**	**Title**	**Venue**
**1**	O. Belo; H. Correia; P. Rodrigues; R. Barros	2016	A personalization system for data visualization platforms	INTECH
**2**	Ü. Aksu; A. del-Río-Ortega; M. Resinas; H. A. Reijers	2019	An Approach for the Automated Generation of Engaging Dashboards	OTM
**3**	O. Belo; P. Rodrigues; R. Barros; H. Correia	2014	Restructuring Dynamically Analytical Dashboards Based on Usage Profiles	ISMIS
**4**	T. Palpanas; P. Chowdhary; G. Mihaila; F. Pinel	2007	Integrated model-driven dashboard development	ISF
**5**	D. Strugar	2020	Recommender systems: Metric suggestion mechanisms applied to adaptable software dashboards	ESEC/FSE
**6**	M. Kintz; M. Kochanowski; F. Koetter	2017	Creating user-specific business process monitoring dashboards with a model-driven approach	MODELSWARD

### RQ1: Have tailoring capabilities ever been applied to performance dashboards in the past?

To answer this research question, we looked at the context that the solutions were applied in. This is crucial because tailored dashboard have been used in a variety of domains, such as Business Intelligence (BI), Internet of Things (IoT), Learning Analytics (LA), and others. We also looked for remarks that indicate whether a proposed solution can be transferred to other domains or contexts. As mentioned previously, we are not only interested in tailored dashboards for performance monitoring, but others that are, according to the authors easily transferable to other domains, in our case performance monitoring.

Belo et al. (2014) presented an analytical system able to dynamically restructure the organisation and contents of its dashboards taking into account usage patterns. The paper proposes an architecture with three layers to achieve this: gathering, storage and management, and restructuring layer. They specifically used this method for cloud-based data warehouses. However, the underlying system for recommendations is general and is based on a user-defined model. Following this observation, it is clear that their personalization system is relevant to the context of performance dashboards.

Pauwels et al. (2009) provided an overview of both the development process and further research considerations for marketing dashboards. Marketing dashboards, judging my the pool of metrics they present, are almost identical to performance ones. In addition, the author clearly state that metrics can be added, edited, or removed easily. However, we consider their five-staged development process to be outdated due to its inability to react to changing requirements and goals of modern organizations. Their adaptation mechanism is based on manual requirement engineering, but it still qualifies as a tailoring mechanism as it is able to evolve over time, as long as newer requirements are added.

Kintz, Kochanowski & Koetter (2017) proposed a model-driven, role-specific process monitoring methodology. It enables users to generate needed information for each user in the organization based on their role. To the best of our knowledge, solution presented in this article is the most mature. Not only is the tailoring mechanism implemented and tested, but they evaluated their solution with a use-case from the service industry. Kintz et al. also made a remark that their approach is easily portable to other use cases, given that new users have that necessary knowledge to send data and model descriptors to their system. However, as mentioned in Vázquez-Ingelmo, Garcia-Peñalvo & Therón (2019a), the resulting dashboard can not be modified at run-time, unlike some of the approaches mentioned previously (Belo et al.).

Belo et al. (2016) presented a multi-agent system for dashboard generation and restructuring that they implemented in the context of cloud-based data warehouses, but the metrics and data sources could be changed and the authors mentioned that other domains could benefit from this approach. They suggest having agents such as the ones for data visualization, profiling evaluation and personalization, and multidimensional data access. This way the entire data flow is covered—from data retrieval to visualization.

Aksu et al. (2019) presented a way to automatically generate engaging dashboards, with a set of KPIs with attributes and their values given as inputs. For their use case they manually created a decision model that decides on the type of chart or graph to be presented on the screen. This manual process would need to be repeated for the context of performance dashboards. However, it would be possible to port their solution to each desired domain.

Strugar (2020) described a way to use item-based collaborative filtering (CF) to generate metric recommendations in performance dashboards. In addition, this work contains the description on how the author analyzed the domain and design spaces to adapt general CF to this exact context. Ultimately, the user ended up using cosine similarity as a measure of item-to-item similarity.

### RQ2: Which factors should be taken into account when designing tailoring capabilities specifically for performance dashboards (requirement management)?

To answer this research question, we looked at different approaches for metric suggestion and how requirement management was used to produce recommendations.

This question was partly answered by Vázquez-Ingelmo, García-Peñalvo & Therón (2019b) and Vázquez-Ingelmo, Garcia-Peñalvo & Therón (2019a). As part of their review they investigated how existing approaches manage user requirements that allow the system to produce personalized dashboard layouts. They identified ten existing approaches that use persistent storage (files or models) to hold user requirements and make data personalized. The reason why we said that the review by Vázquez et al. only partly answers the research question is that with all these approaches user still needs to select widgets to be displayed. Our aim is to examine only approaches where this process is done automatically.

Belo et al. (2014) use a so called *restructurer* agent to suggest a new configuration of the system based on usage log files. Therefore, the only aspect that their approach considers is user-system interactions (usage profiles). They formed a small database schema that further explains which interactions they are taking into account, some of which are changing display mode of a particular widget, or changing the data displayed in a widget altogether. Belo et al. then suggest a way to discover association rules and make the necessary widget changes.

Pauwels et al. (2009) emphasizes the importance of finding out links between metrics. They argue that metrics alone do not address cause-and-effect links with performance change Wyner (2008). Pauwels et al. add that it is advisable to develop a comprehensive experience-driven theoretical framework for certain use cases. The authors do not mention performance dashboards specifically, but their work suggests that the dashboard needs to relate certain user-defined inputs (budget for marketing, salaries, etc.) to market performance metrics, and finally to financial performance. Since the user hand picks the inputs, it is possible to obtain a traditional performance dashboard at the end.

Kintz, Kochanowski & Koetter (2017) propose a model-driven approach that aims to calculate key performance indicators (KPIs) and fulfill certain defined goals. They extended this approach specifically for the user-role relationship within an organization. The manager of the system is responsible for filling in a table that says whether a user with a certain role should be able to see a certain metric. It is worth noting that their approach does not use any usage profiling and the dashboard layout depends solely on the role the user possesses and the configuration of metric access privileges assigned to these roles.

Since Palpanas et al. (2007) use a model-driven approach that entirely depends on the templates and models that system managers provide. As a consequence, system managers are the ones responsible for optimizing the look and feel of a dashboard when the change is due.

Similar to their prior research, Belo et al. (2016) rely on usage logs to profile users and offer them recommendations for improvement. These user logs contain user-system interactions, such as: periods of greatest activity, most requested data, querying preferences, etc. A specific agent that they called *dashboard restructurer* analyzes the log files and passes the information forward to *data visualizers* that present it on the screen.

Aksu et al. (2019) take advantage of different key performance indicator (KPI) attributes required for visualizing dashboards. They examine different components of KPIs that they think users are going to find relevant and engaging, and choose to present that data specifically. Note however, that their method yields different results only if the input (KPI attribute values) changes.

Strugar (2020) suggested to use a combination of implicit and explicit feedback to generate recommendations. Explicit feedback would be manifested in a form of star-based rating review of metrics, while the implicit one would be storing past user-system interactions such as zooming in on specific metrics, changing date intervals, etc. A combination of the two are factors that would be taken into account for new suggestions.

### RQ3: Given that tailored dashboards are often prone to the cold-start problem, what are some of the approaches and/or ideas to help combat it?

Similar to what happens in recommender systems, tailored dashboards that take into account user-system interactions also face the cold-start problem, as observed by Lika, Kolomvatsos & Hadjiefthymiades (2014). Concretely, if no data about the user or the organization is available the system might not know which metrics are relevant. This topic is not explored in some of the existing reviews that we mentioned previously.

A popular approach is creating a preconfigured dashboard for every new user, as used in Belo et al. (2014) and Kintz, Kochanowski & Koetter (2017). This approach, while being straightforward, often serves as a solid starting point Strugar (2020) and Strugar (2019). Researchers also refer to this method as *precooked* dashboards, noted by Ivanov et al. (2018). Then, when users start interacting with the system the dashboard records these interactions and then processes them, leading to a change in the visual layout of the dashboard.

The approach for selecting the ultimate set of metrics to be displayed introduced by Pauwels et al. (2009) is highly relevant for solving the cold-start problem. Balancing out dashboards’ different uses and purposes such as monitoring performance, planning, and communicating information are crucial for dashboard’s success as an information system.

Belo et al. (2016) used a predefined configuration that is forwarded to one of the visualizer agents. This initial configuration is what determines the look of the dashboard, including the metrics to be displayed, at the beginning. This effectively solves the cold-start problem.

Strugar (2020) suggested to use precooked dashboards to generate the initial layout and metrics distribution. The author, however, does not go into detail on how this would be achieved. They do mention that templates would be put in place and designed beforehand to make a *one size fits all* dashboard that would evolve later to adapt to each user.

### RQ4: How effective would tailored performance dashboards be after the users have used the system for a long period of time?

It would be natural to think that as the users interact more with the system the metrics that get suggested to them are getting increasingly relevant if the system takes into account usage profiles. However, this may not be the case, especially taking into account the ever-changing business goals. We tried finding answers to the question, but unfortunately none of the studies included in the review published any updates on their systems being used actively and consistently.

Only the study of Kintz, Kochanowski & Koetter (2017) demonstrated concrete real-world use of their adaptation mechanism. Still, even though their system is capable of handling a variable number of user types, they evaluated their approach only on two roles. In addition, the system only ran for several months at the time when their paper got published. Palpanas et al. (2007) also mentioned that they deployed their solution but do not mention the time interval that their solution was running in.

## Discussion

A further analysis looked at in which context tailoring solutions were applied in, as potential applications are vast. In our review we included either papers related to performance dashboards, or tailored approaches that the authors explicitly mention can be ported to other domains. The following sections include our observations of the results.

### RQ1: Existing approaches for tailoring capabilities in performance dashboards

The answers to RQ1 present the state-of-the-art approaches used to perform tailoring capabilities that are applicable to performance monitoring. We found none of the approaches to be widely accepted. This could indicate that this research area is relatively unexplored and in our opinion it is worth exploring why. Papers outlining this idea date back to 1991, and then they discarded it, noting that this approach is too demanding and technically challenging. Since then, dashboard design and development has become marginally easier and cheaper, making tailored performance dashboards an idea worth exploring once more, given the benefits they provide.

We found two major ways in which a dashboard can adapt itself to the user: *static* and *dynamic*. Static adaptation characterises a mechanism in which the layout is changed only based on data inputted before the execution of a software system. Examples of static adaptation are model-driven approaches and approaches using structured files, and this approach appears to be the most common one currently. The other adaptation mechanism is a dynamic one, in which the layout gets changed due to certain data gathered during the run-time of the system. The most popular dynamic approach for adaptation is usage profiling where user-system interactions are stored and used to generate personalized changes to the system. It is worth noting that to the best of our knowledge there exist no solutions that combine the two into an approach referred by us as *hybrid*.

Naturally, static approaches do not evolve as the user interacts with the system. They depend solely on the configuration done beforehand. As such, we do not see them as fully utilizing the potential of tailored dashboards. As previous research suggests, very careful planning is required to obtain a relevant set of metrics to display on a dashboard in a way that is actionable and insightful. Having a static preconfiguration is therefore most useful in cases where different groups exist with different responsibilities. An example of this in business are roles: a developer, a project manager, and a CEO would clearly be interested in different metrics and would therefore get different layouts only based on one static aspect—their role.

Dynamic approaches take advantage of the fact that it is possible to efficiently store user-system interactions. It is self-evident that these interactions would be useful to dashboard designers, as they could see which parts of the system are often used, and which are not. In contrast to a static approach, dynamic adaptation could therefore be capable of adjust to each user individually. This drastically improves layout’s fit to an individual person using the dashboard.

Combining the two approaches is likely to bring the best out of both worlds, as not only would the dashboard be able to adapt to a certain requirement group (such as roles) but also individually to every person using the system. As mentioned previously, none of the papers suggest a hybrid approach and validating this hypothesis is a part of our future research agenda. For reference purposes we outlined main aspects of different approaches in Table 2.

**Table 2:** Adaptation approaches and their characteristics.

Static Adaptation
**Definition:** mechanism in which the layout is changed only based on data inputted before the execution of a software system.
**Main Benefit:** tailored metrics to a specific user group (e.g., roles within an organization).
**Shortcomings:** they do not allow users to get personalized suggestions during usage of the system.
**Timing:** recommendations are generated before the execution of the system.
**Examples:** model-driven approaches and approaches using structured files.
**Popularity in Research Literature:** most common, well established and explored.
**Observed Research Gap:** exploring whether more specific user grouping can increase relevance of recommendations
Dynamic Adaptation
**Definition:** mechanism in which the layout gets changed due to certain data gathered during the run-time of the system.
**Main Benefit:** potential increased relevance of metrics while users are using the system.
**Shortcomings:** cold-start problem, overfitting.
**Timing:** recommendations are generated during the execution of the system.
**Examples:** usage profiling.
**Popularity in Research Literature:** relatively unexplored, approaches that do exist have not been tested on longer time intervals.
**Observed Research Gap:** use of nondeliberate user interactions (e.g., heat maps and eye heat maps) as a factor in generating recommendations.
Hybrid Adaptation
**Definition:** mechanism that combines both static and dynamic adaptation.
**Main Benefit:** combining the benefits of both static and dynamic approaches, while solving their shortcomings.
**Shortcomings:** more complicated to implement and maintain.
**Timing:** recommendations are generated using the data from both before and during the use of the system.
**Examples:** N/A.
**Popularity in Research Literature:** to the best of our knowledge hybrid approaches have not been proposed yet.
**Observed Research Gap:** empirically evaluating if it performs better than others using different scales (accuracy, serendipity, coverage, etc.).

### RQ2: Requirement management for performance dashboards

As mentioned previously, there are multiple approaches for implementing the tailoring mechanism in terms of data being used to produce the changes. We also noted that we are mostly interested in approaches that generate recommendations automatically, as mentioned in the previous subsection where we talked about static, dynamic and hybrid approaches.

For the static approach there are different aspects of the user that can be taken into account in the context of performance dashboards. The one that has been already mentioned is the role of the user within an organization, e.g., member of board of directors, top management, senior management, middle management, and operational employees. To the best of our knowledge, role is the only user classification group that has already been used in the past. We are interested in how other, more specific, roles could help in presenting more relevant information. To put this is context, even though two users are in the same user group (e.g., board of directors), they might be interested in vastly different metrics: a managing director is interested in day-to-day functioning of the company, while chairmen are responsible for possible future directions of the company. While these two are similar and fall in the same group, the metrics that matter most to them are vastly different. Presenting one with the metrics of the other results in waste of both time and screen estate. Therefore, exploring whether adding more specific user groups could lead to higher metric relevance is also a future research question that we aim to answer with empirical data.

Dynamic metric suggestion remains relatively unexplored, as we only found two papers mentioning usage profiling as a possible way of achieving dynamic adaptation. Here it is crucial to pinpoint which events are taken into account to generate styling changes. Existing solutions only consider deliberate user actions and change of state in the system. While that may be the most reliable way of knowing what needs changing according to the user, it might not be the only one. As an example we suggest looking at widely popular heat maps popularized by Babicki et al. (2016) that give insight into where goes the attention of users. In addition, it might be useful to track eye heat maps (like in Wang et al. (2014)) for non-mouse and non-trackpad inputs. Our stance is that combining heat maps with user-system interactions(changing filters, dates, hiding widgets) would lead to higher metric relevance for the users. This hypothesis is still to be validated.

Finally, as argued previously, we believe the hybrid approach to be most effective, as it eliminates the shortcomings of each of the two variants. Specifically, a dynamic approach could benefit from the immediate increase in metric relevance that static methods provide, and static adaptation could benefit from the increasing relevance that dynamic approaches provide. This approach has not been tried as far as we could tell by performing this review, and our next steps would most certainly reflect this viewpoint.

### RQ3: Solutions to the cold-start problem

From what we observed performing this literature review, dashboards have been often viewed as stationary software artifacts—a lot of work goes into metric selection planning and positioning. Once done, only minor changes were expected. In times where requirements change early and often, we find this approach not to stand the test of time. Non-agile approaches are proven to be ineffective in software development, as noted in Janes & Succi (2014). Software developed once and not maintained does not suffer from the cold-start problem, as careful planning is put in place to ensure that, at least in the start, optimal results are guaranteed. However, adaptable and tailored dashboards evolve over time from a non-optimal start and are therefore susceptible to the cold-start problem.

Providing a generic dashboard to new users is often a starting point. It is vital to increase the relevance early, as users might not benefit from the dashboard if it does not show relevant metrics early on. Static approaches seem to be very useful here, as new users get put in a requirement group, such as their role, and immediately metrics get adjusted. It is apparent that users would benefit greatly from the precooked set of metrics assigned to their role. Therefore, static approaches drastically improves the initial relevance score, thus partially solving the cold-start problem. It is also worth noting that we did not encounter any other method of mitigating this in the literature.

### RQ4: Using tailored performance dashboards for extended periods of time

Following the discussion in the previous section of meeting the cold-start problem in tailored dashboards, once cold-start problem is solved, the goal of tailoring capabilities is to continuously generate increasingly relevant metrics to the user. From what we already noted, it is apparent that dynamic adaptation is supposed to help achieve this.

Dynamic approaches take into account all interactions that the user has with the system. These interactions help with the personalization mechanisms and potentially contributes to a better relevance over time. We say *potentially* because, as noted previously, none of the existing adaptive dashboards in the literature have been running for an extensive period of time in the production environment.

Our assumption is that dynamic approaches would perform increasingly better over time, as they have an ability to capture users’ interactions with the system. However, this could lead to overfitting, as there is a decrease in bias. Therefore, balancing the bias–variance tradeoff is crucial to making dynamic adaptation perform optimally. Additional measures to avoid overfitting have been tried in recommender systems, mostly for evaluating the performance of suggestions—serendipity and coverage, as outlined in Ge, Delgado-Battenfeld & Jannach (2010). We suggest that these additional non-accuracy metrics get considered as well to make dynamic suggestions even more relevant to the user.

**Table 3:** Research takeaways for researchers and practitioners.

Existing Applications of Tailoring Capabilities to Performance Dashboards
**Practitioners:** The most popular methods for adaptation are static ones, but existing approaches have not been extensively used in practise. No empirical evidence shows how effective they are, but given their ease of development, they should be the most attractive option for individuals and organizations aiming to incorporate tailoring mechanisms to their workflow.
**Researchers:** This is an unexplored field, with very few existing approaches, none of which we found to be widely accepted. Potential gaps include: dynamic adaptation mechanisms and developing effective hybrid approaches.
Requirement Management for Tailored Performance Dashboards
**Practitioners:** The most common method that has been tried and proven to be effective is role-based adaptation, where user’s positions within an organization determines what type of metrics should the user see. In addition, dynamic options such as usage profiling are gaining traction, and here user-system interactions are stored and used to generate dynamic suggestions.
**Researchers:** To the best of our knowledge, the only dynamic adaptation method that has been tried in the past is usage profiling. However, existing approaches cover only internal program state changes, and do not take into consideration website heat maps. Exploring how these external factors influence the suggestion process could be an interesting area for exploration.
Existing Approaches for Solving the Cold-Start Problem
**Practitioners:** The only way of solving the cold-start problem practitioners tried in the past are precooked dashboards, where a predefined set of metrics is displayed to an end user.
**Researchers:** Since the only option that has been tried in the past are precooked dashboards, exploring whether the cold-start problem could be practically mitigated by any other method would increase possible implementation options. This could potentially improve the initial user metric suggestion relevance score.
Using Tailored Performance Dashboards for Extended Periods of Time
**Practitioners:** None of the tailored performance dashboards have been reported to run for extended periods of time, and therefore there is not enough past data to suggest that they either effective or ineffective in the long run.
**Researchers:** Given the numerous benefits tailored performance dashboards bring to the table, exploring how viable they are in the long run could either drastically improve or worsen their popularity and use in practise. Analyzing the empirical data and making statistical claims about performance scores is the suggested way of tackling this problem.

### Summary of the state of the art with respect to the research questions

Table 3 provides an easy to reference takeaways separated by research questions for both theoretical and practical dashboard designers. It contains four sections, corresponding to each of the four research questions we outlined previously.

The first section of the table draws attention to currently employed approaches and which of them is most popular and practical for use at this instant. The second one deals with requirement management and the use of different mechanisms to achieve tailoring functionalities. The third section explains how the cold-start problem is solved today, and remarks that there really has been only one widespread solution. Finally, the fourth section aims to emphasize that no solutions have been used for bigger time frames and that more data is needed to either support or reject the hypothesis that they perform better than traditional options in the long run.

## Threats to Validity

First, as this review suggests, the area of adaptable performance dashboards remains relatively unexplored. This is why we decided to include some papers that are not necessarily about tailored performance dashboards. The reason for inclusion was that the authors explicitly mentioned that the solutions are easily transferable to different domains.

Second, literature on performance dashboards dates back to early 1990s. Since then, no major reviews have been published that evaluate their effectiveness and technical considerations. Considering that web technology has improved drastically in the last thirty years, both feasibility as well as performance aspects of such papers were not examined. Technologies used back then are obsolete now and we were unable to even reproduce their results.

Third, our entire review process assumed a standard common knowledge of newly introduced terms, search methods, and analysis methods. As we wanted to avoid bias, potential misunderstandings were mitigated by having a reviewed shared document outlining the detailed illustrations of the search and review processes, as well as the review results.

Lastly, we evaluated our systematic review against a predefined set of four widely accepted quality questions proposed by Kitchenham & Charters (2007).

## Conclusions

We conducted a systematic literature review to study existing approaches of tailoring mechanisms for performance dashboards. Our focus was on primary studies published in peer-reviewed journals and conferences.

Our study results indicate that not many methods were proposed, and especially tried for an extended period of time in real industrial scenarios. As the area is getting traction lately as noted by Vázquez-Ingelmo, García-Peñalvo & Therón (2019b), we recommend the following to make studies more attractive to practitioners:

1. To obtain the unbiased relevance score we advise researchers to empirically evaluate the effectiveness of their proposed approaches using metrics like accuracy, serendipity, and coverage.
2. To allow others to understand why a particular tailoring mechanism was used, details on research design and methodology should be provided, in addition to the credibility assessment of authors’ findings.
3. Since dashboards are used continuously it is vital to evaluate how the adaptability mechanisms perform in the long run, say months after being first deployed. If data on this is not readily available, the authors should consider discussing the potential future performance of their proposed systems.
4. Even though dynamic and hybrid adaptation is unexplored and there are only a handful of solutions already proposed, future findings should explain how their solutions mitigate the cold-start problem and avoid overfitting.

We used the results of this review to form a classification based on the nature of tailoring approaches, taking into account the type of data, and timing of recommendations. The resulting classification is shown in the Discussion section and it captures different aspects of these mechanisms such as their benefits, shortcomings, adoption, and directions for future research.

Based on study findings, we suggest the following as possible future research opportunities:

1. Research on the impact of more specific user grouping (e.g., their role, responsibilities, tasks, etc.) on metric relevance.
2. Research on the impact of nondeliberate user interactions (e.g., heat maps and eye heat maps) on relevance score of dynamic/hybrid approaches.
3. Research on how long-term dashboard use affects relevance score in dynamic/hybrid approaches.
4. Research on the optimal ordering and structuring of widgets to form a display of information available to users at a glance.
5. Research on domain adaptation and how existing solutions can be ported to the domain of performance dashboards and vice versa.

To sum up our findings and make contributions clear and useful, we created Table 3 to contain insights for both practitioners and researchers.

## Supplemental Information

10.7717/peerj-cs.625/supp-1 Supplemental Information 1 Comprehensive summary of the data to support analysis and replications

10.7717/peerj-cs.625/supp-2 Supplemental Information 2 Code used to perform the analysis of data to support analysis and replications

## Additional Information and Declarations

### Data Availability

The following information was supplied regarding data availability: The data and code are available in the Supplementary Files.
